# Development and Characterization of Extrudates Based on Rapeseed and Pea Protein Blends Using High-Moisture Extrusion Cooking

**DOI:** 10.3390/foods10102397

**Published:** 2021-10-09

**Authors:** Izalin Zahari, Ferawati Ferawati, Jeanette K. Purhagen, Marilyn Rayner, Cecilia Ahlström, Amanda Helstad, Karolina Östbring

**Affiliations:** 1 Department of Food Technology Engineering and Nutrition, Lund University, Naturvetarvägen 12, 22362 Lund, Sweden; jeanette.purhagen@food.lth.se (J.K.P.); marilyn.rayner@food.lth.se (M.R.); cecilia.ahlstrom@food.lth.se (C.A.); amanda.helstad@food.lth.se (A.H.); karolina.ostbring@food.lth.se (K.Ö.); 2 Malaysian Agricultural Research and Development Institute (MARDI), Persiaran MARDI-UPM, Serdang 43400, Selangor, Malaysia; 3 Department of Chemistry and Biomedical Sciences, Linnaeus University, 39231 Kalmar, Sweden; ferawati.ferawati@lnu.se

**Keywords:** meat analogs, high-moisture extrusion, extrudates, texturized protein, plant-based protein, rapeseed protein concentrate, pea protein isolate

## Abstract

Rapeseed protein is not currently utilized for food applications, although it has excellent physicochemical, functional, and nutritional properties similar to soy protein. Thus, the goal of this study was to create new plant-based extrudates for application as high-moisture meat analogs from a 50:50 blend of rapeseed protein concentrate (RPC) and yellow pea isolate (YPI) using high-moisture-extrusion (HME) cooking with a twin-screw extruder to gain a better understanding of the properties of the protein powders and resulting extrudates. The effects of extrusion processing parameters such as moisture content (60%, 63%, 65%, 70%), screw speed (500, 700, and 900 rpm), and a barrel temperature profile of 40–80–130–150 °C on the extrudates’ characteristics were studied. When compared to the effect of varying screw speeds, targeted moisture content had a larger impact on textural characteristics. The extrudates had a greater hardness at the same moisture content when the screw speed was reduced. The specific mechanical energy (SME) increased as the screw speed increased, while increased moisture content resulted in a small reduction in SME. The lightness (L*) of most samples was found to increase as the target moisture content increased from 60% to 70%. The RPC:YPI blend was equivalent to proteins produced from other sources and comparable to the FAO/WHO standard requirements.

## 1. Introduction

There has been a growing recognition that extra effort must be taken to shift the global consumption of proteins towards more plant-based alternatives. This is owing to increased global populations and limited natural resources, making animal protein production an increasingly unsustainable approach [1]. Furthermore, environmental and ethical concerns regarding animal husbandry, as well as health concerns over red meat, are prominent issues of animal-based proteins [2]. Attention has therefore been given to the nutritional quality of new and alternative protein sources. For this reason, researchers are investigating various protein sources based on their physical and chemical properties, functional properties, and nutritional value, including amino acid composition [3,4,5,6,7].

Meat is a complex product that is appreciated for its flavor and texture. Thus, mimicking meat is technologically challenging. Lentil, soy, wheat, and fungal proteins are common examples of plant proteins used in the production of meat analogs. Several texturizing techniques have been investigated, including extrusion cooking [2,4,8]. Extrusion cooking technique is a high-temperature, short-time, adaptable, and contemporary food operation which turns granular or powdered agricultural commodities into completely cooked food items with improved texture and taste qualities [2,9]. Extrusion cooking refers to the continuous mixing, shearing, heating, and shaping of a composition utilizing single or twin screws within a heated barrel. Twin-screw extruders offer good conveying performance, allowing the handling of formulas with intermediate viscosity and higher moisture content (>40%). The food material to be extruded is conveyed through the barrel towards the die section before it cools and forms a solid strand. The protein-rich materials are sheared and cooked in the heated and pressurized barrel, facilitating protein unfolding and crosslinking [10]. Because of the processing versatility it offers, extrusion cooking has become a mainstay in the food industry, particularly in meat products [10], cereal [11], dairy [12], pasta [13], flour [14], and pet food areas [15].

Alternative protein sources from plants are increasingly being investigated due to sustainability and product economy reasons. To provide a sufficient supply of protein for the world’s population, a high-protein crop alternative is needed to fulfill the rising global demand. Soy protein has mostly been used as a meat substitute because of its outstanding features and high-quality protein. Rapeseed (*Brassica napus*) and yellow pea (*Pisum sativum*) are readily cultivated in Europe and could become an alternative to soybean. Rapeseed was the second most prevalent oilseed crop in the world after soybean in 2019, according to the Food and Agriculture Organization (FAO). Rapeseed is commercially cultivated largely for its oil content and favorable fatty acid composition [16]. However, the primary interest in both rapeseed and yellow pea has been in the animal feed industry and aquaculture industries [17]. Only a small fraction of these protein-rich crops are used in the human diet, such as cooking oil for rapeseed and fresh or cooked pea. In the production of rapeseed oil, a press cake (if cold-pressed) or rapeseed meal (if hot-pressed) is generated, which contains around 38–45% high-quality protein [17]. Of the total protein contained in rapeseed, ~60% of the proteins are cruciferin (11S globulin), and 25–45% are napin (2S albumin) [18]. Oil body proteins and lipid transfer proteins are minor proteins present in the seed, with oleosins constituting the majority (75–80%) of oil body proteins in rapeseed [18,19]. Meanwhile, in pea, albumin and globulin represent 10–20% and 70–80% of the total seed protein, respectively [20]. Rapeseed protein is also renowned for having a well-balanced amino acid composition [21,22], as well as indications that the proteins are technologically useful [23,24,25]. All these factors point to rapeseed meal being a viable source of high-quality protein for use in the food processing sector. In our previous study, meat analogs were successfully developed from hemp protein concentrate, which could substitute soy protein isolate up to 80% [26]. Numerous studies on rapeseed have been conducted in the past, including isolation processes, anti-nutritional component reduction, physicochemical and functional characteristics investigation, as well as nutritional aspects [21,22,23,24,27,28,29,30,31,32]. In the late 1970s, Kozlowska et al. [31] studied the structures of textured plant protein preparations such as flour and concentrates derived from soybean and rapeseed, as well as blends made by extrusion-cooking rapeseed concentrate and soybean flour in a 1:1 ratio under high- and low-pressure conditions. The results revealed that the high-pressure method yields a product with a specific use as a meat extender, whereas the low-pressure procedure yields a product appropriate for the production of meat analogs.

The aims of this study were (i) to investigate the physicochemical characteristic of the rapeseed protein concentrate in comparison to the commercial yellow pea isolate and the mixture of both protein (50:50); and (ii) to process the rapeseed–yellow pea protein-rich materials into extrudates for application as high-moisture meat analogs.

## 2. Materials and Methods

### 2.1. Materials

Cold-pressed rapeseed press cake (*Brassica napus* L.), industrially produced by Gunnarshögs Gård AB (Hammenhög, Sweden), was used as the protein source for protein isolation, while commercial yellow pea isolate, YPI (80% protein, 3% carbohydrate, 6% fat, wet basis) was obtained from Bulk Powders Company, Colchester, UK. Other chemicals used for the isolation process were food-grade and obtained from VWR International, Stockholm, Sweden. Both protein powders were mixed at 50:50 ratios before being used for further analysis and extrusion cooking.

### 2.2. Isolation Protocol of Rapeseed Protein Concentrate

Rapeseed protein concentrate was prepared from rapeseed press cake on a semi pilot scale. A total of 2 kg rapeseed press cake was dry-milled using a Robot Coupe food processor (R302V, Atlanta, Georgia) at room temperature at 2000 rpm for 2 min. The pulverized press cake was mixed with water (1:10 *w*/*w*), and the pH was adjusted to 10.5 with 2 M NaOH, based on the previous study [33]. Then, the rapeseed meal was extracted by stirring (IKA RW 28 digital, Germany) for 1 h at room temperature while maintaining the pH. Thereafter, the slurry was separated using a decanter with a 56 mm weir disc (Decanter Centrifuge DM80, Lemitech GMBH, Germany) at 2000× *g* and differential screw speed of 10 rpm. A peristaltic pump was used to set the inflow to 20 L/h (Masterflex Easy-load Model 77200-62, Cole-Parmer, Vernon Hills, IL, USA). It took approximately 1 h to complete the decanting process before the slurry phase was taken to the next step. Citric acid was added to adjust the pH to the isoelectric point (pI) at 3.5 [34], and the slurry was stored at 4 °C overnight. The stored liquid separated into two phases: one bottom phase containing the precipitated proteins, and one clear top phase containing the non-precipitated components. The clear top phase was decanted by a Masterflex pump, and the bottom phase was centrifuged at 4700× *g* for 20 min (Beckman Coulter, Allegra^®^X-15R Centrifuge, Brea, CA, USA) to recover the precipitated protein concentrate. The protein concentrate was diluted with tap water 1: 1 water (*w*/*v*), the pH was adjusted to 7 with 2 M NaOH, and was agitated at ambient temperature. The protein concentrate was freeze-dried in a vacuum freeze dryer (Epsilon I/30; Martin Christ, Osterode, Germany) at 40 Pa and a plate temperature of −60 °C. The dried protein concentrate was milled and stored at −18 °C prior to analysis and extrusion processing.

### 2.3. Analysis of Protein Powders

#### 2.3.1. Proximate Composition

Moisture content was determined by drying the samples in an oven at 105 °C for 16 h, in accordance with the standard methods of AOAC 934.0 [35]. Crude protein was determined by the Dumas combustion method using a protein analyzer (Flash EA 1112 Series, Thermo Scientific, Waltham, MA, USA), according to AOAC 990.03, and a conversion factor of 6.25 was used to calculate the total protein content [36]. Total fat contents were measured by solvent extraction using petroleum ether solvent in accordance with AOAC 920.39 in the semi-automatic Soxtec equipment (Tecator AB, Höganäs, Sweden) [37]. Ash and crude fiber content were determined according to AOAC 923.03 and AOAC 991.43, respectively [38,39]. All analyses were performed in triplicate. Carbohydrate content was calculated by difference.

#### 2.3.2. Bulk Density

The bulk density (g/mL) was measured by gently pouring 2 g of protein powder into an empty 10 mL graduated cylinder and tapping 10 times on a rubber pad from a height of 15 cm. The bulk density is determined by the mass of the powder divided by the volume of the cylinder [40].

#### 2.3.3. Water- and Oil-Holding Capacity

For water-holding capacity (WHC) and oil-holding capacity (OHC), 1 g of protein powder was transferred into centrifuge tubes, and 10 g of distilled water or oil was applied, respectively. The resulting suspensions were vortexed at high speed for 2 min before being centrifuged for 30 min at 3000× *g* [41]. The supernatant was discarded, and the weight of the resulting sediment was calculated. The following equation was used to express the water retention and oil absorption capacities:

WHC or OHC = (W2 − W1)/W1 (1)

where W1 is the mass of the dry sample and W2 is the mass of the obtained gel-like sediment (hydrated or oil-based paste). All determinations were conducted on triplicate samples.

#### 2.3.4. Particle Size Measurement

Particle size measurement was determined by a sieving technique where five sieves with descending opening mesh were mounted on top of each other in a sieve shaker (J. Engelmann, Ludwigshafen, Germany) based on the standard AACC method 66-20.01 [42]. The sieves used had the following mesh sizes: 246 µm, 175 µm, 147 µm, 125 µm, and 74 µm. Every sample was shaken for 5 min, and the different powder fractions were collected and weighed. The percent retained on each sieve was calculated. The cumulative percent of powder retained on each sieve was determined by adding up the total amount of powder that was retained on each sieve and the amount in the previous sieves.

#### 2.3.5. Pasting Properties

The pasting properties of RPC, YPI, and the mixed protein powder were measured by a slightly modified version of the standard AACC method 76-21.02 using a Rapid Visco Analyzer 4800 (Perten Instruments, Perkin Elmer, NSW, Australia) [43]. The samples were prepared by mixing the sample (3.50 g) with water (26.66 g) at 14% moisture basis as recommended by the manufacturer’s instruction, heated to 50 °C, and stirred under a constant shear rate at 960 rpm for 10 s. The slurry was held at 50 °C for 50 s and then heated up to 130 °C, with a temperature increase of 12 °C/min. It was held at 130 °C for 2.5 min and finally cooled to 50 °C at 12 °C/min. The pasting properties of each raw material and mixtures were measured in duplicate at least.

#### 2.3.6. Thermal Properties

The thermal properties of RPC and YPI were determined using a Differential Scanning Calorimetry (DSC) instrument (Seiko Instruments Inc.-EXSTAR6000 DSC, Shizuoka, Japan), calibrated with indium, and an empty pan was used as a reference. A total of 2 mg of protein powder was weighed using a precision balance (+0.01 mg) into a coated aluminum pan, and MilliQ water was added three times the weight of the sample. The pan was sealed and heated at a rate of 10 °C/min from 25 °C to 160 °C. Each sample was tested in duplicate, and the data were saved and analyzed with DSC software (SII EXSTAR6000 Muse, Shizuoka, Japan). Based on the dry weight of the samples, the melting temperature and enthalpies were computed from the thermograms [26].

#### 2.3.7. Amino Acid Analysis

Due to the limitation of sample materials, the amino acid composition was performed on RPC and YPI. The mix was therefore calculated based on the results from the pure powders. Amino acid profiles of the protein concentrate/isolate samples were determined at Eurofins Food & Feed Testing Sweden using the standardized method (ISO 13903:2005, EU 152/2009) with an amino acid analyzer [44]. Samples were hydrolyzed with 6 M HCL, and amino acids were separated by ion-exchange chromatography and determined by post-column reaction with ninhydrin, using photometric detection at 570 nm and 440 nm.

#### 2.3.8. Evaluation of Amino Acid Composition

The contents of different amino acids recovered were presented as g/100 g protein and were compared with the FAO/WHO (2013) reference pattern [45]. The ratio of essential to total amino acids was reported as E/T (%):

(2) $$\frac{E}{T}\%=\frac{\mathrm{Ile}+\mathrm{Leu}+\mathrm{Lys}+\mathrm{Met}+\mathrm{Cys}+\mathrm{Phe}+\mathrm{Tyr}+\mathrm{Thr}+\mathrm{Val}+\mathrm{His}}{\mathrm{Ala}+\mathrm{Asp}+\mathrm{Arg}+\mathrm{Gly}+\mathrm{Glu}+\mathrm{Ile}+\mathrm{Leu}+\mathrm{Lys}+\mathrm{Met}+\mathrm{Cys}+\mathrm{Phe}+\mathrm{Tyr}+\mathrm{Thr}+\mathrm{Val}+\mathrm{His}}\times 100$$

The amino acid score (*AAS*) was calculated by the method of FAO/WHO as shown below:

(3) $$AAS=\frac{\mathrm{mg}\;\mathrm{of}\;\mathrm{AA}\;\mathrm{in}\;1\;g\;\mathrm{of}\;\mathrm{test}\;\mathrm{protein}}{\mathrm{mg}\;\mathrm{of}\;\mathrm{AA}\;\mathrm{in}\;1\;g\;\mathrm{the}\;\mathrm{FAO}/\mathrm{WHO}\;\mathrm{reference}\;\mathrm{pattern}}\times 100\;$$

### 2.4. High-Moisture Extrusion Cooking

For extrusion cooking, rapeseed protein concentrate was milled into fine particles using a Robot Coupe food processor (R302V, Atlanta, GA, USA) before being mixed with the commercial yellow pea isolate protein at 50:50 ratios. The mixture of protein materials was mixed well with a mixer equipped with a double whisk (Bosch Universal Plus, München, Germany) before being fed into a laboratory co-rotating twin-screw extruder (KETSE 20/40D, Brabender GmbH & Co.KG, Duisburg, Germany) with a clamshell barrel opening system.

**Figure A1:**
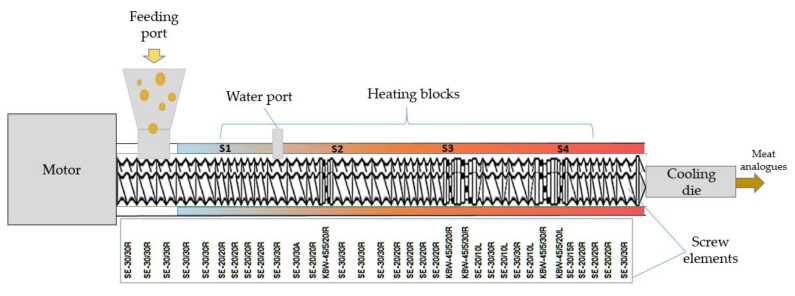
Schematic illustration of high-moisture extrusion (HME) with twin-screw extruder (KETSE 20/40D, Brabender GmbH and Co., Duisburg, Germany) and the screw configuration. S1, S2, S3 and S4 = heating sections in barrel, SE = screw element, KBW = kneading block.

The operational extruder parameters are shown in Table 1. The extruder was comprised of four barrel sections for temperature control and adjustment. The screw diameter (D) was 20 mm, and the whole configured screw length (L) was 40D. The operating screw speeds used in this study were 500, 700, and 900 rpm. The temperature in each barrel section was held constant at 40–80–130–150 °C. Based on our previous research, a similar screw configuration was employed, with the selected temperatures being the optimal combination that may give an acceptable texture for the extrudates formulated with an oilseed and/or legume protein mix [26,46]. The screw arrangement was created using a mix of feeding, conveying, compression, and kneading components (Appendix A, Figure A1). The mixture was metered into the feed port at a rate of 3 kg/h (dry basis) using a loss-in-weight volumetric gravity feeder (Feeder Control Module Congrav OP, Brabender GmbH & Co.KG, Duisburg, Germany). During extrusion, water was introduced directly into the feeding zone using a pump to maintain the targeted moisture content. From previous trials, four moisture levels were selected for this study, which were 60%, 63%, 65%, and 70% (Appendix B, Table A1. The protein extrudate was formed into a rectangular strip as it exited the cooling die (the internal dimension of the die was 7 mm × 25 mm × 300 mm), and the pressure at the die was recorded using a pressure transducer. The Brabender screw configuration software (WinExt-Software, Brabender GmbH & Co.KG, Duisburg, Germany) was used for documentation and archiving, including collecting extruder parameter data at 1 s intervals. The data and extrudate samples were collected when the torque and pressure operating conditions had stabilized at each new set of operation conditions, which took around 8 min. Texture properties were determined on the extrudates (Section 2.5), and the samples were thereafter kept in a sealed plastic bag and stored at −18 °C until further analysis.

The specific mechanical energy (SME; kJ/kg), which is defined as the amount of work supplied from the driving motor into the raw material being extruded, was used to quantify extrusion process characteristics [47]. The SME was calculated according to the following equation [48]:

(4) $$\mathrm{SME}\;(\mathrm{kJ}/\mathrm{kg})=\frac{2\pi \times n\times T\;}{\mathrm{MFR}}$$

where n is the screw speed (rpm), *T* is the torque (Nm), and MFR is the mass flow rate (kg/h).

### 2.5. Texture Properties

Texture properties were measured on the same day as the extrusion trial. The overall texture of the extruded products was evaluated by texture profile analysis (TPA) and cutting tests using a texture analyzer (TVT-300XP, Perten Instruments AB, Hägersten, Sweden). For TPA, samples with a size of 20 mm × 20 mm and a thickness of 7 mm were compressed by 2 mm using a cylindrical probe (18 mm). The samples’ hardness, springiness, resilience, and chewiness were measured and calculated. Two kinds of cutting resistance, transverse and longitudinal, were tested for the cutting test. A knife blade (height 117 mm) was used to test the cutting strength by piercing the sample (20 mm, 7 mm thickness) to a depth of 5 mm at a speed of 2 mm/s. The blade was always wider than the size of samples to ensure symmetry. Transversal cutting was carried out in the direction of the sample width, while longitudinal cutting was conducted in the direction of the sample length [26].

### 2.6. Color Determination and Visual Appearance

The color of the protein powder and the produced extrudates was measured using a colorimeter (Konica Minolta CR-400, Osaka, Japan). Calibration was performed with a white calibration tile. The parameters of the CIE-Lab were expressed as L* (lightness), a* (redness to greenness), and b* values (yellowness to blueness). For each sample, the measurements were performed in triplicate at randomly chosen locations. In order to generate high-quality images of the produced extrudates, images were acquired by a mounted camera (Nikon D3300, AF-P DX 18-55/3.5-5.6G, Nikon Company, Tokyo, Japan) in a photo box with black walls and four light sources illuminating at the sample.

### 2.7. Statistical Analysis

All experiments were analyzed using MINITAB 16 software and Microsoft Office Excel 2010. The significance of the results was performed using a one-way analysis of variance (ANOVA), and Tukey’s test was performed to verify the statistical significance of each sample at *p* < 0.05, with a 95% confidence level.

## 3. Results and Discussion

### 3.1. Proximate Composition

The proximate composition and functional properties of RPC, YPI, and the mixture (50:50) are listed in Table 2. The proximate composition of rapeseed press cake is also included for comparison. The moisture content of RPC, YPI, and mixed protein were 1.7%, 6.8%, and 4.0%, respectively. The difference in values between both pure RPC and YPI is due to the different drying techniques used, which were freeze-drying for RPC and spray-drying for YPI. Because of the extended drying duration, which can range from several hours to several days under vacuum, freeze-drying is known to yield low moisture levels in samples. Spray-drying, on the other hand, is a rather rapid process with a drying time of a few seconds to a minute and results in moisture levels of 7% [49]. The protein content in RPC was found to be lower (56.2%) than that of commercial YPI protein content (82.3%), while the mixed protein content is 69.9%. RPC had a greater fat content (23.7%) than YPI (0.4%), which might contribute to favorable properties in meat analog products [50]. The fat level of the rapeseed press cake resulted in the high content of fat in RPC due to the formation of a lipid–protein complex [51]. The reason for this also can be explained by the technique of oil extraction utilized. As previously reported [33], the cold-pressed rapeseed cake utilized in this study has a comparatively high fat content compared to hot-pressed rapeseed meals. High values of ash in both protein powders may indicate that the materials are good sources of minerals.

### 3.2. Bulk Density, Water- and Oil-Holding Capacity

**Figure 1:**
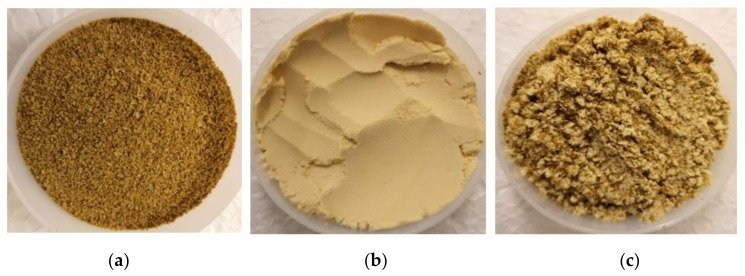
Appearance of three different protein powders: (**a**) RPC; (**b**)YPI; (**c**) mixed protein (50:50).

The results of bulk density and water- and oil-holding capacity are presented in Table 2. There was no significant difference in the bulk density of all three samples. YPI powder had a higher water-holding capacity compared to RPC. On the other hand, the oil-holding capacity of RPC was greater than that of YPI, which was likely due to the presence of rapeseed oleosin, which is known to interact with and assist the stabilization of oil droplets in the seed [52]. The low value of YPI indicates the existence of a high fraction of hydrophilic compounds as opposed to hydrophobic groups on the surface of the protein molecules. Figure 1 depicts the appearance of the protein powders. It can be clearly seen that RPC powder is coarser than YPI powder, which might be owing to the drying technique utilized, as previously stated. Spray-drying generated a finer powder, while the freeze-dried product was processed using a miller, resulting in larger particles. Figure 1 also shows that RPC powder had a dark brown color, whereas YPI powder was light yellow in color. Depending on the pH used throughout processing and temperatures involved in the final drying process, the hues of protein powder might vary, notably from light tan to dark brown for rapeseed. This might be related to the phenolic oxidation and protein–phenolic interaction during the leaching in alkali conditions during the protein-isolation process [18].

### 3.3. Particle Size Measurement

**Figure 2:**
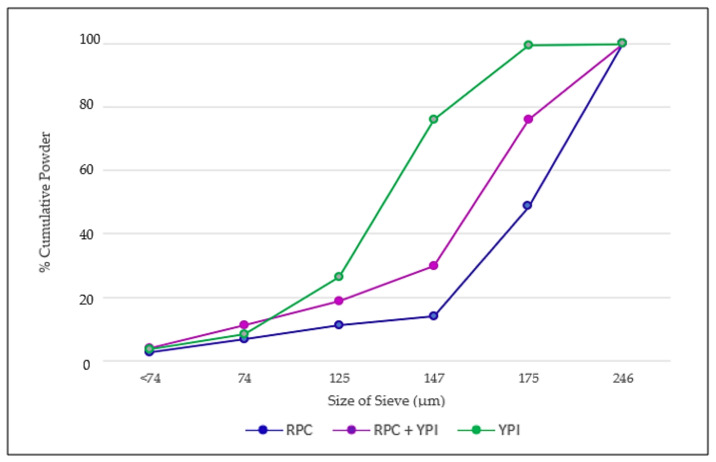
Graphical representation of the percentage of cumulative powder at the different sizes of sieves for all three different protein powder samples.

Table 3 shows the result of the particle size measurement using a sieving technique performed on various samples of RPC, YPI, and the mixed powder. The significant differences (*p* < 0.05) in the mass of particles that remained in the sieve were observed at the range of x > 246 μm and 147 < x ≤ 175 μm. Sieve fractionation indicated that more than 50% of the RPC particles were >175 µm, whereas more than 50% of YPI and mixed powder particles were >125 µm and >147 µm in size, respectively (Figure 2). According to the result of the experiment, YPI has a smaller particle size than RPC. This result was in line with the results of the above-mentioned water- and oil-holding capacity tests, which were obviously impacted by the varied processing techniques.

### 3.4. Pasting Properties

**Figure 3:**
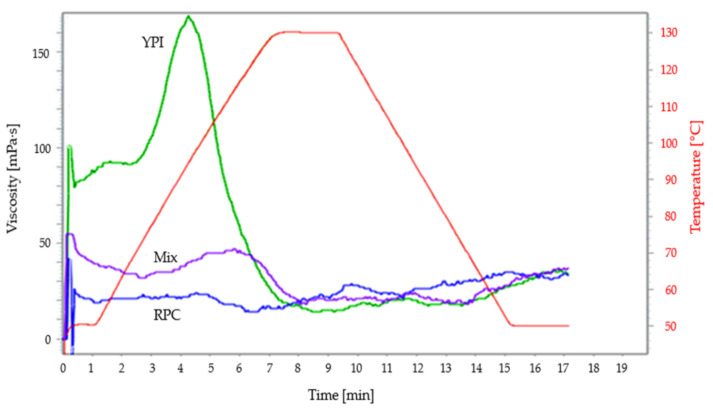
Pasting properties for YPI, RPC, and mixed proteins.

Figure 3 illustrates the pasting properties of three different protein powders. YPI samples exhibit cold swelling in the beginning, followed by an increased viscosity upon heating. YPI powder showed the highest value of peak viscosity (165.5 ± 4.9 mPa·s), followed by RPC (55.5 ± 37.5 mPa·s), and mixed protein (47.5 ± 0.7 mPa·s). The consequence of the difference in the peak viscosities of the protein powder, according to a previous study, is that such protein might behave differently during cooking due to various rates of water absorption by the sample [53]. The breakdown value of RPC, YPI, and the mixed proteins were 41, 153, and 27 mPa·s, respectively. Because the YPI samples had larger breakdown viscosities, it suggests that their proclivity to produce a paste with relatively increased instability during cooking is quite high [54]. The high viscosity curve of YPI at 130 °C indicates the denaturation of the native protein at that particular temperature. Mixing two proteins had a noticeable influence on the pasting characteristics, which might be due to the varied structure of the protein gel network produced; thus, differences in texture variability in the extrudates can be expected. In this study, the final viscosity ranged from 28 to 38 mPa·s, which was a small difference between RPC and YPI. The peak time for protein materials used ranged from 4.2 to 7.5 min, which describes the time taken to achieve the peak viscosity while heating at 130 °C [55].

### 3.5. Thermal Properties

Table 4 shows the DSC thermogram results of two distinct protein samples, revealing the temperature required and the magnitude of these changes. RPC had two endothermic peaks, while YPI had three. Protein denaturation occurs when bonds that are important in the creation and maintenance of the protein structure are disrupted by thermal energy [56]. The peaks with the highest onset temperature (peak 2 for RPC and peak 3 for YPI) also had the highest transition enthalpy, which indicates that these are the main denaturation temperatures. Furthermore, the magnitude of the RPC enthalpy was higher than for the YPI, indicating that denaturation is occurring to a greater extent. This could be a result of the gentle extraction process used for RPC, leaving more protein in its native state before DSC analysis. For RPC, there were reasonably large peaks at around 98.9 °C and 132.9 °C, which may be the denaturation temperatures corresponding to cruciferin and napin, respectively [29,57]. Those temperatures were, however, slightly higher than those previously reported for pure cruciferin and napin, which are 91 °C and 110 °C [58]. According to Perera et al. [58], the cruciferin structure unfolds at pH 3 at ambient temperatures. The RPC in the present study was precipitated at pH 3.5, which could explain the denaturation temperature deviations from the literature. It was also reported that many of the protein molecules in the resulting rapeseed protein products are acid-denatured to some extent, while napins are reported as hydrophilic proteins, which remain stable at temperatures as high as 75–100 °C [57,59]. According to Wu and Muir [57], a variety of variables may influence the thermal stability of rapeseed protein isolates, including protein structure, amino acid content, metal and other prosthetic group bindings, intramolecular interactions, protein–protein contacts, linkages, and environmental conditions.

For YPI, three peaks were observed in this study at 68.8, 97.6, and 130.9 °C, which corresponds to the reported thermal denaturation of vicilin (7S) and legumin (11S) fractions, in accordance with results from previous studies [46,60]. Due to their heterogeneity, these proteins account for various endotherms and variations in denaturation temperature [60].

### 3.6. Amino Acid Composition and Evaluation

The amino acid composition of all protein powders including the 50:50 mixtures (by calculation), is shown in Table 5, and the FAO/WHO (2013)-suggested requirements of the essential amino acids for older children, adolescents, and adults are also included. In terms of essential amino acids, RPC contains high values of threonine, histidine, and tryptophan, while YPI exceeds all the requirements. Although RPC has lower values in other amino acids (lysine, valine, isoleucine, and leucine), YPI can compensate by increasing those amino acids to meet the standard requirement. All protein powders had higher total aromatic amino acid (phe + tyr) content than that of FAO/WHO. RPC compensated for the lower content of sulfur-containing amino acid in YPI (Table 5), although it is still lower than the standard requirement. However, the values of sulfur-containing amino acids in the mixed protein is still higher than other plant sources reported previously, such as in hempseed protein isolates (1.39%), soy protein isolates (0.92%), and whole flaxseed extracts (1.31%) [27]. All protein powders were also rich in glutamic acid (8.47–13.50 g/100 g protein) and aspartic acid (4.87–9.74 g/100 g protein), which was in agreement with previous reports [27]. According to Hou et al. [61], non-essential amino acids have been proposed in many studies to impact not only the taste and flavor of food but also the growth and health of animals and humans. Based on the calculation, most of the amino acids in the protein mixture sample met the FAO/WHO requirement. According to Osen et al. [8], there was no significant reduction of amino acids (*p* > 0.05) observed in the pea extrudates under high-moisture extrusion. This result was supported by several findings [62,63], which showed that the high moisture reduces the shear stress and dissipation of mechanical energy in the extruder (compared to low-moisture extrusion), and thus could protect the loss of amino acids during extrusion. The total essential amino acids of the mixed protein were almost equivalent to the FAO/WHO standard, which indicates that the mixed protein had a good nutritional value. E/T ratios for all protein samples were around 40%, which was deemed acceptable [45]. Taken together, the amino acid profile of RPC:YPI mixed powder is comparable to proteins derived from other sources, such as soy and milk, and met the FAO/WHO standard requirement. According to the amino acids score (*AAS*) in Table 6, the first limiting amino acid in RPC is lysine, while the second limiting amino acid is leucine. Fledderman et al. [64] also found lysine to be the first limiting amino acid in rapeseed protein isolate. Monsour et al. [65], on the other hand, identified valine as the first limiting amino acid in rapeseed protein concentrate. As for YPI and the mixed protein, met + cys and valine are the first and second limiting amino acids found in this study. The high pH, lengthy processing time, genetic and environmental (geographical) variations have all been observed to alter the amino acid content of rapeseed, according to several studies [27,28].

### 3.7. High-Moisture-Extrusion Cooking

**Figure 4:**
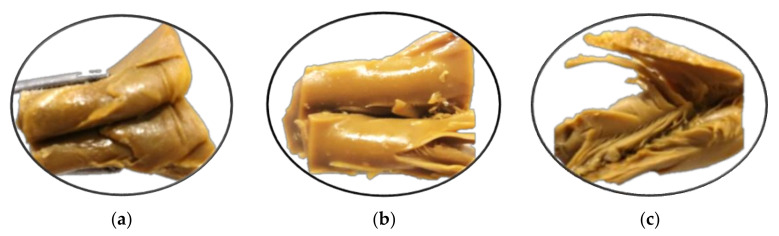
Fibre structure of extrudates from RPC:YPI mixture at 900 rpm and different target moisture content: (**a**) 60% MC; (**b**) 63% MC; (**c**) 65% MC.

Extrudates with significant layered fibrous structures were successfully produced from the RPC:YPI mix at all levels of target moisture content (60%, 63%, 65%, 70%), screw speeds (500, 700, and 900 rpm), and extrusion temperatures of 40–80–130–150 °C. Overall, the texture of the extrudates generated was found to be softer at higher moisture levels (70%) and firmer at lower target moisture contents (60%) at all screw speeds examined. However, when the moisture level of the extrudates increased, the layered features of the extrudates exhibited more fibrous structural arrangement, as demonstrated in Figure 4, which is consistent with previous studies [66]. Increased screw speed, on the other hand, resulted in a more prominent fibrous structure in the extrudates. SME and pressure are known to be impacted by a drop in slurry temperature when the feed is pushed through the cooling feed channel, as well as an increase in viscosity when protein–protein interaction and crosslinking occurs [67,68]. As expected, the SME rises as the screw speed increases; however, increasing target moisture content resulted in a small drop in SME due to a reduction in shear force and mechanical energy input (Table 7). Others have previously reported similar findings [66,69]. However, between 63% and 65% at 500 and 900 rpm, and likewise between 63% and 70% at 700 rpm, there was no significant difference (*p* < 0.05) found.

### 3.8. Texture Properties

The textural properties of extrudates from the RPC:YPI mixed extruded at various target moisture contents and screw speeds are shown in Table 7. Hardness is a measurement of how hard the product is and can be determined by the maximum force of the first compression [70], and chewiness was defined as the amount of energy required to chew the extrudates [71]. Overall, compared to the effect of varied screw speeds, moisture content level had the largest influence on instrumentally assessed textural attributes, particularly the considerable reduction (*p* < 0.05) of the hardness and chewiness values as the moisture content increased. Lowering the screw speed, on the other hand, resulted in increased extrudate hardness and chewiness at the same moisture level. Lower screw speed increased the residence time within the extruder barrel, allowing a higher shearing impact on the melt, which in turn could have resulted in increased creation of new bonds and improved texturization. Springiness shows how well the product returns to its original structure after the first compression, while resilience describes how well the product regains its original height after the compression [70]. In this study, the chewiness and springiness were determined to be greatest in 60% MC at 700 rpm and in 65% MC at 500 rpm, respectively. The springiness of the extrudates ranged from 0.84 to 0.94. Furthermore, altering the screw speed had no influence on springiness or resilience, with the exception of the latter at 70% moisture content. These findings were consistent with other results from other plant-based extrudates reported previously [72].

**Figure 5:**
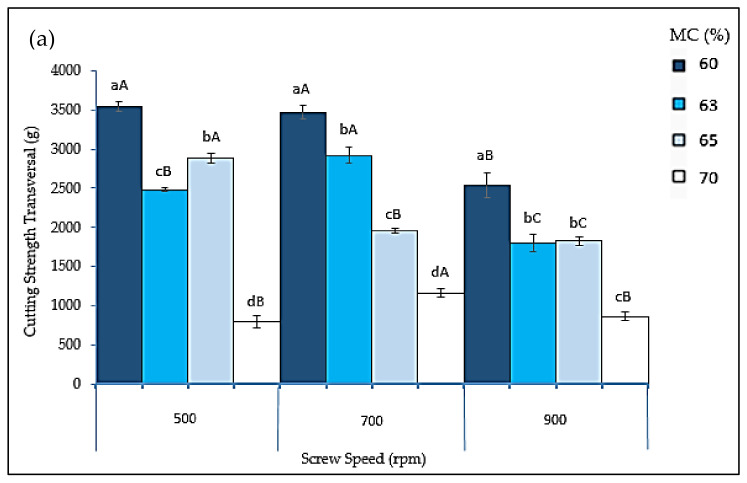

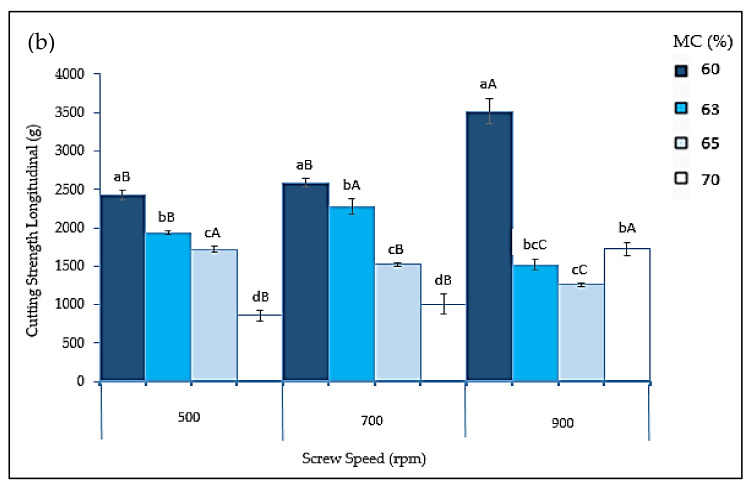
(**a**) Transversal cutting strength; (**b**) longitudinal cutting strength of different extrudates formulations at each target moisture content. Bars represent the mean ± standard deviation. Different lowercase letters indicate a significant difference between different target moisture contents at the same screw speed, and different uppercase letters indicate a significant difference between different screw speeds at the same target moisture content (Tukey’s test, *p* < 0.05). MC = moisture content.

The cutting strength in both transversal and longitudinal directions was inversely proportional to the moisture content in the extrudates, as can be seen in Figure 5. The highest cutting strength was found for extrudates with the lowest target moisture content investigated (60%) at all levels of screw speed tested, while the lowest cutting strength was achieved in the materials with the highest target moisture content (70%), with the exception at 900 rpm for the longitudinal direction. This might be due to the combination of low viscosity and temperature of the melt in the barrel when more moisture was added, resulting in incomplete protein denaturation and hence reduced protein interaction [73]. Furthermore, the meat analogs’ transversal cutting strength was somewhat greater than their longitudinal cutting strength values, as previously observed for the texture of extruded soy protein isolate [72]. Our findings also revealed that, with the exception of 900 rpm, there was a significant difference (*p* < 0.05) between various target moisture contents in both cutting strength directions of the extrudates generated.

### 3.9. Color Determination and Visual Appearance

**Figure 6:**
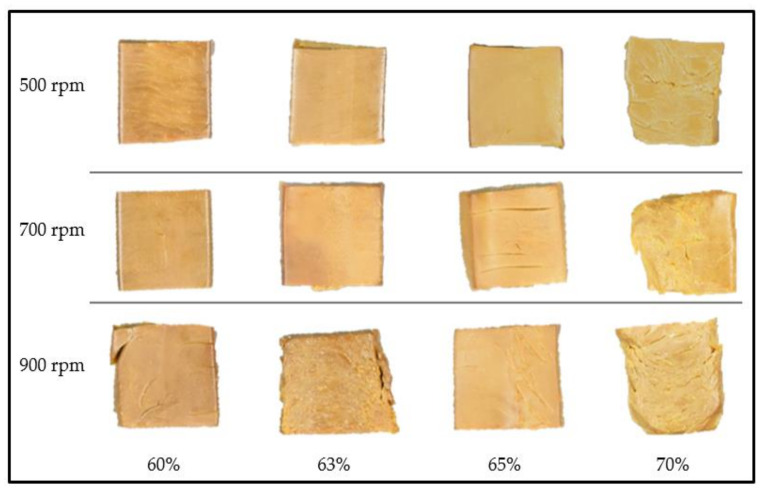
The visual appearance of the extrudates from mixed proteins (RPC: YPI) at different screw speeds and different target moisture contents.

Color attributes (lightness, redness, and yellowness) of the extrudates at three different screw speeds and four levels of target moisture content are presented in Table 8. As the target moisture content was increased from low (60%) to high (70%) during extrusion, lightness (L*) of all samples was found to increase except for 63% moisture content at 500 rpm. On the other hand, all samples showed no significant differences (*p* < 0.05) of lightness at the same target moisture content while varying the screw speed except at 65 and 63%. The same trend was reported previously, where higher moisture content led to lighter meat analogs during high-moisture-extrusion cooking [73]. The higher L* values for the extrudates were a result of lower rates of chemical reactions in the protein composite processed with higher water content. According to Santellán-Moreno et al. [74], changes of color during the extrusion process might be due to Maillard reactions, caramelization, hydrolysis, and pigment degradation. Berset [75] stated that color changes could also be a sign of the process’s intensity, which can be linked to chemical changes. Notably, most of the samples showed significant differences (*p* < 0.05) in yellowness (b*) between the moisture levels at each specific screw speed. For visualization, Figure 6 shows the appearance of each extrudate in this study. The dark brown color of extrudates produced from the mixed proteins, in comparison to the color of animal flesh, may not be a major concern for imitating the color of meat. However, a sensory evaluation would be required to determine the perception and acceptability of consumers towards the texturized protein created.

## 4. Conclusions

This study is the first step towards a deeper understanding of extrudates from rapeseed and pea protein using high-moisture-extrusion. Overall, texturized plant protein extrudates were successfully developed from rapeseed protein concentrate and yellow pea protein at 50:50 ratios. The mixed protein powder with about 70% protein content was extruded at different target moisture contents (60%, 63%, 65%, 70%) and screw speeds (500, 700, 900 rpm) using fixed barrel segment temperatures (40–80–130–150). As predicted, the extrusion temperature and screw speed should be set at a higher level to ensure denaturation of the protein in the mixed powder; thus, a sufficient fiber formation during high-moisture-extrusion could be achieved. The moisture level was shown to be more important than the screw speed in affecting the texture of the extrudates. Varying the target moisture content would impact both cutting strength directions. Our study also revealed that increasing the screw speed required more energy, but that will decrease when the moisture content increases. The mixed protein powder, as well as the extrudates, provided promising qualities in terms of chemical composition, amino acid composition, color, and texture properties. The appearance of the extrudates, in combination with the texture, functionality, and nutritional content, could help the mixed powder of rapeseed and pea protein acquire market acceptability. Our study is a contribution to the knowledge needed to tackle one of the most pressing problems in the near future: an adequate supply of protein in a sustainable manner.

## Figures and Tables

**Table 1:** Operational extruder parameters used in the study.

Extrusion Parameters	Values
Power	12 kW
Operating screw speed	500, 700, 900 rpm
Screw diameter	20 mm
Screw length	800 mm (40D)
Feed rate	3 kg/h
Barrel temperature	40, 80, 130, 150 °C
Solid dosing	0.94–1.25 kg/h
Liquid dosing	1.75–2.06 kg/h

**Table 2:** Chemical characterization and functional properties of RPC, YPI, and the mixed protein.

Parameter	Rapeseed Press Cake	RPC	YPI	Mixed Protein
Moisture (%)	10.3 ± 0.1	1.7 ± 0.2	6.8 ± 0.0	4.0 ± 0.1
Protein (%)	27.3 ± 0.1	56.2 ± 0.4	82.3 ± 0.7	69.9 ± 0.9
Fat (%)	15.5 ± 0.1	23.7 ± 0.1	0.4 ± 0.2	11.2 ± 0.2
Ash (%)	6.04 ± 0.1	6.3 ± 0.1	4.0 ± 0.1	5.1 ± 0.0
Carbohydrate, by difference (%)	10.8	12.1	6.5	9.8
Crude fiber (%)	30.1 ± 1.5	<1.0 ± 0.15	<1.0 ± 0.15	n.d.
Bulk density (g/mL)	n.a.	0.51 ± 0.0	0.52 ± 0.0	0.52 ± 0.0
Water-holding capacity (mL/g)	n.a.	1.74 ± 0.01	3.99 ± 0.02	2.76 ± 0.03
Oil-holding capacity (mL/g)	n.a.	1.24 ± 0.01	0.59 ± 0.02	1.02 ± 0.01

Each value is the mean (% wet basis) ± standard deviation.

**Table 3:** Particle size measurement details of three different protein powder samples.

Sieve Diameter (µm)	Particle Size (µm)	RPC Particle (g)	YPI Particle (g)	Mixed Protein Particle (g)
<74	0 < x ≤ 74	1.17 ± 0.33 ^a^	1.71 ± 1.04 ^a^	1.88 ± 0.82 ^a^
74	74 < x ≤ 125	1.99 ± 0.98 ^a^	2.12 ± 1.10 ^a^	3.45 ± 2.26 ^a^
125	125 < x ≤ 147	2.17 ± 1.27 ^a^	8.50 ± 4.22 ^a^	3.58 ± 1.34 ^a^
147	147 < x ≤ 175	1.38 ± 1.92 ^a^	23.12 ± 5.63 ^b^	5.14 ± 2.73 ^a^
175	175 < x ≤ 246	16.48 ± 3.77 ^a^	10.94 ± 8.94 ^a^	21.91 ± 3.88 ^a^
246	x > 246	24.59 ± 1.43 ^a^	0.15 ± 0.02 ^b^	11.34 ± 0.19 ^c^
Amount of sieved powder (g)	n.a	47.78 ± 3.21	46.54 ± 5.97	47.30 ± 4.71
Sieved powder (%)	n.a	99.31	99.43	99.85

Each value is the mean ± standard deviation. Different lowercase letters within rows indicate a significant difference (Tukey’s test, *p* < 0.05) between different protein particles.

**Table 4:** Transition temperatures of RPC and YPI.

	RPC		YPI		
	Peak 1	Peak 2	Peak 1	Peak 2	Peak 3
**Onset Temperature, T_o_ (°C)**	95.7 ± 4.4	129.5 ± 2.5	64.7 ± 2.1	95.4 ± 2.8	127.9 ± 0.8
**Peak Temperature, T_p_ (°C)**	98.9 ± 5.6	132.9 ± 2.1	68.8 ± 3.1	97.6 ± 1.7	130.9 ± 1.6
**Conclusion Temperature, T_c_ (°C)**	101.8 ± 2.8	134.7 ± 3.5	72.6 ± 0.0	104.3 ± 2.3	132.3 ± 1.7

Each value is the mean ± standard deviation.

**Table 5:** Amino acid composition (g/100 g protein) of RPC, YPI, and mixed protein, and the FAO/WHO-suggested requirements (adult) of essential amino acids.

Amino Acid	RPC	YPI	Mixed Protein *	Older Child, Adolescent, Adult Daily Requirement **
Threonine ^a^	2.59	2.98	2.79	2.30
Methionine ^a^	1.14	0.82	0.98	
Phenylalanine ^a^	2.58	4.24	3.41	
Histidine ^a^	1.49	2.00	1.75	1.50
Lysine ^a^	2.86	6.07	4.47	4.50
Valine ^a^	3.04	4.03	3.54	3.90
Isoleucine ^a^	2.50	3.66	3.08	3.00
Leucine ^a^	4.51	6.69	5.60	5.90
Tryptophan ^a^	0.93	0.73	0.83	0.60
Cysteic acid	0.86	0.76	0.81	
Tyrosine	1.96	2.87	2.42	
Serine	2.54	4.42	3.48	
Glycine	2.98	3.15	3.07	
Glutamic acid	8.47	13.50	10.99	
Proline	2.81	3.51	3.16	
Alanine	2.61	3.32	2.97	
Arginine	3.70	6.73	5.22	
Aspartic acid	4.87	9.74	7.31	
Total sulfur-containing amino acids (Met + Cys)	2.00	1.58	1.79	2.20
Total aromatic amino acids (Phe + Tyr)	4.54	7.11	5.83	3.80
Total essential amino acids	21.64	31.22	26.45	27.70
Total non-essential amino acids	31.73	48.73	40.26	
E/T (%)	41.27	39.41	40.15	

All values are expressed in g of amino acid per 100 g of protein. ^a^ Essential amino acid. * Mixed protein by calculation. ** WHO/FAO/NUO adult indispensable amino acid requirements pattern [FAO, 2013]. E/T = the proportion of essential amino acids to the total amino acids.

**Table 6:** Amino acid scores of three different protein powders. (a) *AAS* of RPC, YPI, and mixed protein (50:50).

Amino Acids Score (*AAS*)	RPC	YPI	Mixed Protein
Thr	112.61	129.57	121.30
His	99.33	133.33	116.67
Lys	63.56 ^a^	134.89	99.33
Val	77.95	103.33 ^b^	90.77 ^b^
Ileu	83.33	122.00	102.67
Leu	76.44 ^b^	113.39	94.92
Tryp	155.00	121.67	138.33
Met + Cys	90.91	71.91 ^a^	81.36 ^a^
Phe + Tyr	119.47	187.11	153.42
Total EAA	78.69	113.53	96.18

^a^ The first limited amino acid. ^b^ The second limited amino acid.

**Table 7:** Texture profile analysis result of extrudates from mixed protein (RPC:YPI) at different screw speeds and target moisture contents.

Screw Speed (rpm)	MC (%)	SME (kJ/kg)	Texture Attributes			
			Hardness (g)	Springiness	Resilience	Chewiness (g)
500	60	503 ± 0 ^a^	6715 ± 17 ^aA^	0.84 ± 0.02 ^aA^	0.61 ± 0.01 ^aA^	5698 ± 8 ^aA^
	63	457 ± 16 ^b^	5807 ± 208 ^bA^	0.93 ± 0.07 ^aB^	0.57 ± 0.01 ^bA^	4832 ± 157 ^bA^
	65	453 ± 0 ^b^	4865 ± 71 ^cA^	0.94 ± 0.06 ^aA^	0.49 ± 0.01 ^cA^	3922 ± 81 ^cA^
	70	412 ± 21 ^c^	1850 ± 135 ^dA^	0.88 ± 0.02 ^aA^	0.47 ± 0.02 ^cAB^	1501 ± 117 ^dA^
700	60	859 ± 30 ^a^	6714 ± 33 ^aA^	0.93 ± 0.06 ^aA^	0.63 ± 0.02 ^aA^	5716 ± 50 ^aA^
	63	816 ± 36 ^b^	3736 ± 161 ^bB^	0.88 ± 0.01 ^aA^	0.52 ± 0.08 ^abA^	3467 ± 399 ^bB^
	65	774 ± 0 ^c^	3677 ± 81 ^bB^	0.87 ± 0.02 ^aA^	0.50 ± 0.03 ^bA^	3114 ± 190 ^bA^
	70	824 ± 34 ^b^	694 ± 188 ^cB^	0.90 ± 0.09 ^aA^	0.51 ± 0.02 ^bA^	537 ± 133 ^cB^
900	60	1267 ± 0 ^a^	4166 ± 276 ^aB^	0.87 ± 0.06 ^aA^	0.62 ± 0.03 ^aA^	3464 ± 391 ^aB^
	63	1213 ± 47 ^b^	2857 ± 92 ^bC^	0.87 ± 0.06 ^aA^	0.55 ± 0.03 ^abA^	2343 ± 69 ^bB^
	65	1195 ± 38 ^b^	1664 ± 24 ^cC^	0.90 ± 0.09 ^aA^	0.52 ± 0.01 ^bA^	1278 ± 97 ^cB^
	70	1276 ± 29 ^a^	1083 ± 157 ^dB^	0.89 ± 0.11 ^aA^	0.43 ± 0.04 ^cB^	837 ± 131 ^cB^

All values are presented as mean ± standard deviation. Different lowercase letters indicate a significant difference between different target moisture content at the same screw speed, and different uppercase letters indicate a significant difference between different screw speeds at the same target moisture content (Tukey’s test, *p* < 0.05). MC = moisture content, SME = specific mechanical energy.

**Table 8:** Result of L*, a*, and b* of extrudates from mixed proteins (RPC:YPI) at different screw speeds and target moisture contents.

Screw Speed (rpm)	Target Moisture Content (%)	Color Parameters		
		L*	a*	b*
500	60	34.99 ± 0.60 ^cA^	3.55 ± 0.28 ^abB^	14.43 ± 0.36 ^cC^
	63	39.19 ± 0.17 ^bA^	3.53 ± 0.04 ^abB^	19.28 ± 0.46 ^aB^
	65	38.95 ± 0.23 ^bB^	3.82 ± 0.12 ^aB^	18.64 ± 0.25 ^aC^
	70	40.82 ± 0.72 ^aA^	3.35 ± 0.01 ^bB^	17.49 ± 0.10 ^bA^
700	60	36.21 ± 0.52 ^cA^	3.95 ± 0.02 ^bAB^	17.40 ± 0.15 ^cB^
	63	38.07 ± 0.41 ^bB^	3.92 ± 0.06 ^bA^	21.19 ± 0.42 ^aA^
	65	40.69 ± 0.09 ^aA^	4.56 ± 0.10 ^aA^	19.86 ± 0.16 ^bB^
	70	41.18 ± 0.30 ^aA^	3.67 ± 0.12 ^cA^	16.15 ± 0.35 ^dB^
900	60	35.23 ± 0.43 ^cA^	4.15 ± 0.10 ^aA^	19.48 ± 0.11 ^cA^
	63	36.98 ± 0.48 ^bC^	4.03 ± 0.04 ^aA^	20.14 ± 0.18 ^bB^
	65	41.05 ± 0.27 ^aA^	3.55 ± 0.02 ^bC^	20.66 ± 0.02 ^aA^
	70	41.52 ± 0.09 ^aA^	3.36 ± 0.13 ^bB^	17.48 ± 0.16 ^dA^

All L*, a*, b* values are presented as mean ± standard deviation. Different lowercase letters indicate a significant difference between different target moisture content at the same screw speed, and different uppercase letters indicate a significant difference between different screw speeds at the same target moisture content (Tukey’s test, *p* < 0.05).

## Data Availability

The datasets generated for this study are available on request to the corresponding author.

## Appendix B

**Table A1:** Selected processing variables used for extrusion of the mixed proteins.

Raw Material	Barrel Segment Temperatures (°C)				Target Moisture Content (%)	Screw Speed (rpm)
	S1	S2	S3	S4		
RPC: YPI mix (50:50)	40	80	130	150	60	500 700 900
	40	80	130	150	63	500 700 900
	40	80	130	150	65	500 700 900
	40	80	130	150	70	500 700 900
